# Metabolomic Profiling of the Synergistic Effects of Melittin in Combination with Cisplatin on Ovarian Cancer Cells

**DOI:** 10.3390/metabo7020014

**Published:** 2017-04-14

**Authors:** Sanad Alonezi, Jonans Tusiimire, Jennifer Wallace, Mark J. Dufton, John A. Parkinson, Louise C. Young, Carol J. Clements, Jin-Kyu Park, Jong-Woon Jeon, Valerie A. Ferro, David G. Watson

**Affiliations:** 1 Strathclyde Institute of Pharmacy and Biomedical Sciences, University of Strathclyde, Glasgow G4 0RE, UK; alonezi-sanad-mohammed-z@strath.ac.uk (S.A.); jonanstusiimire@must.ac.ug (J.T.); Louise.c.young@strath.ac.uk (L.C.Y.); c.j.clements@strath.ac.uk (C.J.C.); v.a.ferro@strath.ac.uk (V.A.F.); 2 Department of Pharmacy, Faculty of Medicine, Mbarara University of Science and Technology, P.O. Box 1410 Mbarara, Uganda; 3 WestCHEM Department of Pure and Applied Chemistry, University of Strathclyde, Glasgow G1 1XL, UK; jennifer.wallace.101@strath.ac.uk (J.W.); mark.dufton@strath.ac.uk (M.J.D.); john.parkinson@strath.ac.uk (J.A.P.); 4 #204, Beesen Co. Ltd., Bio Venture Town, Yuseong Daero 1662, Dae Jeon 34054, Korea; jkypark@live.co.kr (J.-K.P.); confessor@hanmail.net (J.-W.J.)

**Keywords:** melittin, cisplatin, synergy, sensitive and resistant ovarian cancer cells, metabolomics

## Abstract

Melittin, the main peptide present in bee venom, has been proposed as having potential for anticancer therapy; the addition of melittin to cisplatin, a first line treatment for ovarian cancer, may increase the therapeutic response in cancer treatment via synergy, resulting in improved tolerability, reduced relapse, and decreased drug resistance. Thus, this study was designed to compare the metabolomic effects of melittin in combination with cisplatin in cisplatin-sensitive (A2780) and resistant (A2780CR) ovarian cancer cells. Liquid chromatography (LC) coupled with mass spectrometry (MS) was applied to identify metabolic changes in A2780 (combination treatment 5 μg/mL melittin + 2 μg/mL cisplatin) and A2780CR (combination treatment 2 μg/mL melittin + 10 μg/mL cisplatin) cells. Principal components analysis (PCA) and orthogonal partial least squares discriminant analysis (OPLS-DA) multivariate data analysis models were produced using SIMCA-P software. All models displayed good separation between experimental groups and high-quality goodness of fit (R^2^) and goodness of prediction (Q^2^), respectively. The combination treatment induced significant changes in both cell lines involving reduction in the levels of metabolites in the tricarboxylic acid (TCA) cycle, oxidative phosphorylation, purine and pyrimidine metabolism, and the arginine/proline pathway. The combination of melittin with cisplatin that targets these pathways had a synergistic effect. The melittin-cisplatin combination had a stronger effect on the A2780 cell line in comparison with the A2780CR cell line. The metabolic effects of melittin and cisplatin in combination were very different from those of each agent alone.

## 1. Introduction

Combination therapy has long been studied in the treatment of cancer, including ovarian cancer [1]. It is a logical approach, focusing on increasing the response and tolerability to treatment, while also decreasing resistance [2]. Unfortunately, it can be difficult to assess whether a particular combination will behave in a synergistic, additive, or antagonist fashion when used on a particular cancer patient. The only known way of determining their effectiveness is to identify specific measures, such as response rate, survival, or time to progression, and assess if or whether the new combination is able to achieve a significant improvement [2]. Combination therapy is essentially cooperative: each agent involved should have non-overlapping toxicities, different mechanisms of action with minimal cross-resistance, and individually proven in treatment by itself [2]. 

Several cancers, including ovarian cancer, have demonstrated resistance or reduced sensitivity to cisplatin treatment, leading to decreased time to disease progression, increased likelihood of relapse, and reduced efficacy upon re-treatment during relapse [3,4]; cisplatin itself causes significant health problems, such as nephrotoxicity [5]. Ideally, cisplatin in combination with a drug that diminishes its negative effects while enhancing the therapeutic effects would decrease resistance or relapse while mitigating its negative effects. For example, cisplatin (or another platinum agent) in combination with taxanes (e.g., paclitaxel) is now regarded as standard chemotherapy in ovarian cancer, where the taxane enhances the tumour’s radiosensitivity. Cisplatin and the adenovirus OBP-301 have also been shown to work synergistically [1].

Cisplatin shares cytotoxic synergy with bee venom [1]. Bee venom makes sense as a complement to cisplatin: it has protective effects in many areas of the body such as the blood and nerves [6]; it is able to inhibit cell growth in tumours [7]; and has even been examined and used in complementary treatments that are necessitated by the effects of chemotherapy, such as allodynia [8] and neuropathy [9]. Cisplatin in combination with bee venom has been successfully used against human cervical and laryngeal carcinoma cells, including their drug-resistant sublines [10], and human glioblastoma [11]. Several studies have found that phospholipase A_2_, another component of bee venom, not only mitigates the negative impact of cisplatin on kidneys [12], but also boosts regulatory T cell (Treg) numbers in the spleen and enhances Treg traffic to the kidneys following cisplatin exposure. This is important, as Tregs play a significant role in many mechanisms, including inflammation and autoimmunity suppression [4,12,13]. A significant bonus in this dynamic is that bee venom has no negative impact on the anti-cancer properties of the cisplatin [4], meaning it in no way diminishes the effect of the cisplatin treatment. This is critical; a combinatory agent which diminishes any positive effects of the primary treatment undermines its effectiveness overall. The effects that cisplatin and bee venom have together on ovarian cancer cells emerge from the synergistic relationship between the two agents [14]. Alizadehnohi et al. report that separately and together, the two agents induce apoptosis in human ovarian cancer cells; bee venom appears to enhance the cytotoxic impact of cisplatin [14]. It also appears that melittin provokes responses in cisplatin-sensitive cells which lead to decreasing levels of amino acids, which in turn affect the energy metabolism of the tumour [15].

In order to understand these effects and the metabolic changes that the cisplatin-melittin combination has on ovarian cancer cells, metabolomic investigations can be undertaken. Metabolomics in the context of oncology usefully focuses on diagnosis and prognosis, as well as on evaluating the effectiveness of therapy [16,17]. For instance, one study employed nuclear magnetic resonance (^1^H-NMR) spectroscopy and was able to accurately separate serum metabolite profiles of three groups of patients: namely women with ovarian cancer, normal premenopausal women, and women with a benign ovarian disease [18]. In addition, LC-MS in combination with Biolog Microarray assays revealed that treatment of cisplatin-resistant and sensitive ovarian cancer cells with melittin distinctively altered their lipid profiles and their ability to metabolise certain carbon energy sources, [15]. NMR spectroscopy has also been used to produce and examine metabolic profiles in other cancers such as hepatocellular carcinoma [19].

In essence, the effects of the cytotoxic mechanisms of both cisplatin and melittin, together and separately, cause changes in cells which can be identified and measured through metabolomic analysis. The current study aimed to examine the metabolic effects of melittin in combination with cisplatin on A2780 and A2780CR human ovarian cancer cell lines using a LC-MS based metabolomics approach employing a ZIC-pHILIC column. Multivariate data analysis was performed based on PCA and OPLS-DA models constructed using the SIMCA-P software.

## 2. Results

### 2.1. The Cytotoxicity of Melittin in Combination with Cisplatin

**Figure 1:**
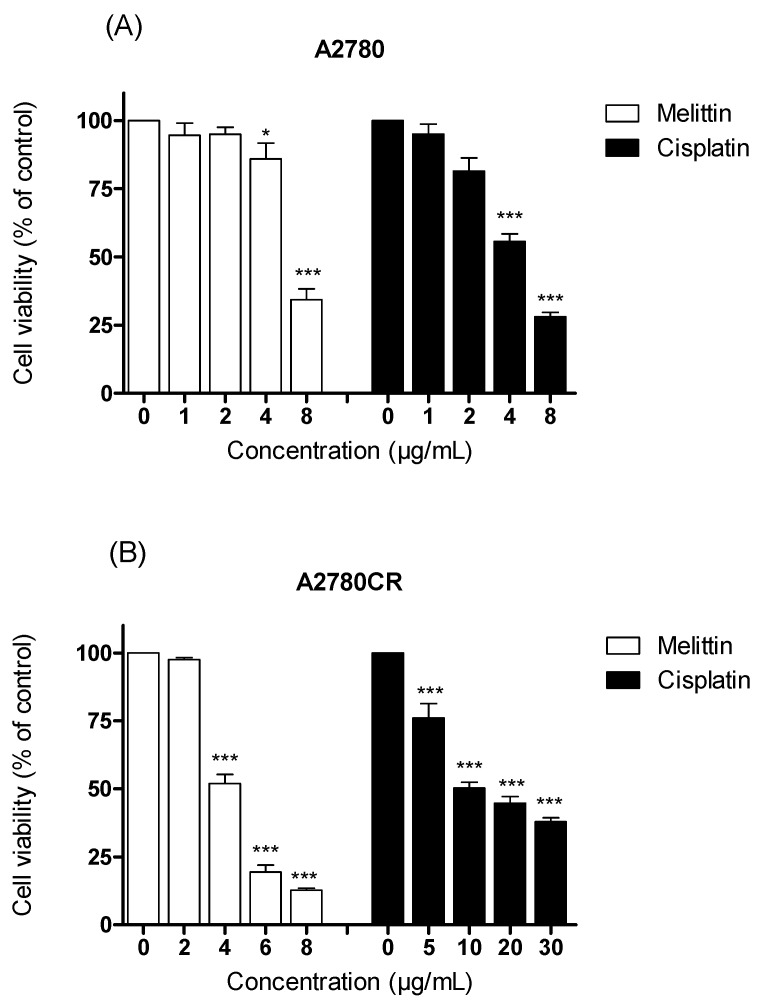
Examination of cell viability after treatment with either cisplatin or melittin alone with various concentrations on (**A**) A2780 and (**B**) A2780CR cell lines. * Significantly different from zero. concentration at <0.05; *** Significant at <0.001.

**Figure 2:**
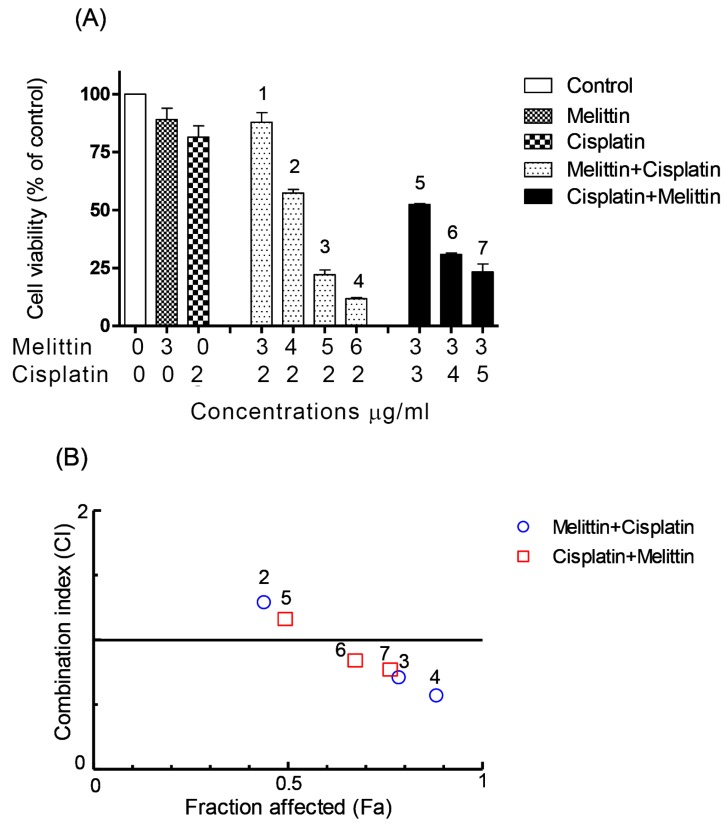
Effects of melittin in combination with cisplatin on (**A**) cell viability of A2780 cell lines and (**B**) combination index. (**A**) The A2780 cells were treated with various concentrations of the melittin + cisplatin combination for 24 h. Bar graphs represent mean ± SD values. (**B**) Combination index (CI) analysis was generated using the method of Chou and Talalay [21,22] to determine the extent of synergy, if any, for the melittin + cisplatin combination on A2780 cell lines.

**Figure 3:**
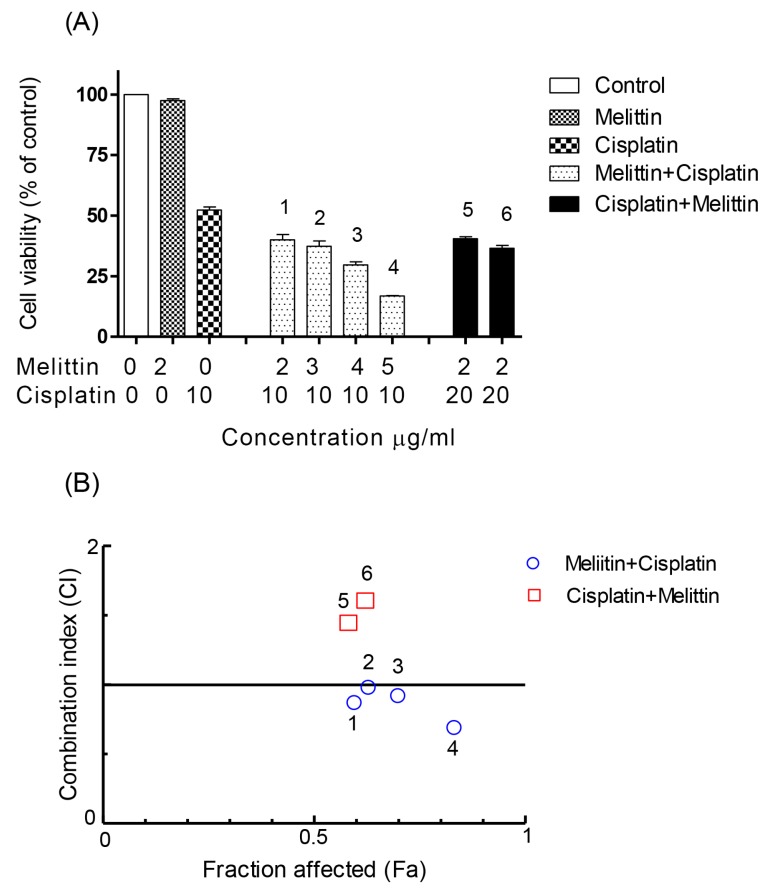
Effect of melittin in combination with cisplatin on (**A**) cell viability of A2780CR cell lines and (**B**) combination index. (**A**) The A2780CR cells were treated with various concentrations of the melittin + cisplatin combination for 24 h. Bar graphs represent mean ± SD values. (**B**) Combination index (CI) analysis was generated using the method of Chou and Talalay [21,22] to determine the extent of synergy, if any, for the melittin + cisplatin combination on A2780CR cell lines.

The viabilities of the cisplatin-sensitive and cisplatin-resistant ovarian cancer cells (OCCs) (A2780 and A2780CR, respectively) treated with melittin or cisplatin were compared. Both single treatments exerted a concentration-dependent cytotoxic effect on A2780 and A2780CR cells (Figure 1). The cisplatin mediated growth inhibition of the sensitive cell line (A2780) was significantly greater than that of the melittin over the concentration range of 1 to 8 μg/mL (Figure 1A; *p* < 0.05). However, the A2780CR cells, as expected, were more resistant to cisplatin than the A2780 cells (Figure 1B). The 24 h half maximal inhibitory concentrations (IC_50_) of cisplatin in A2780CR and A2780 cells were 10.8 and 4.9 μg/mL, respectively. Melittin exhibited toxicity against both A2780CR and A2780 cells, with IC_50_ values of 4.5 and 6.8 μg/mL, respectively [15] (Figures S1 and S2). The cytotoxicity of melittin in combination with cisplatin against A2780 and A2780CR cells was studied by using an Alamar^®^ Blue assay [15]. The A2780 and A2780CR cells were treated for 24 h at various concentrations of melittin in combination with cisplatin. The percentage of surviving cells decreased in a dose-dependent manner in both cell lines. The cytotoxic effects of melittin in combination with cisplatin on A2780 and A2780CR cell lines are shown in Figure 2A and Figure 3A, respectively. 

### 2.2. The Combination Index (CI)

In order to qualitatively evaluate whether the combination of melittin with cisplatin might cause synergistic cytotoxic effects, the value of CI, a commonly used evaluation index, was calculated. It has been proposed that CI values be interpreted as follows: <0.1 very strong synergism, 0.1–0.3 strong synergism, 0.3–0.7 synergism, 0.7–0.9 moderate to slight synergism, 0.9–1.1 nearly additive, 1.1–1.45 slight to moderate antagonism, 1.45–3.3 antagonism, and >3.3 strong to very strong antagonism [20]. 

The CI analyses showed that synergistic cytotoxic activity on A2780 cells occurred with the melittin + cisplatin combinations at concentrations of 5 + 2 (CI = 0.647) and 6 + 2 (CI = 0.512) μg/mL, and with the cisplatin+melittin combinations at concentrations of 4 + 3 (CI = 0.789) and 5 + 3 (CI = 0.711) μg/mL, respectively. However, the calculated CI was found to be >1 in A2780 cells treated with the melittin + cisplatin combinations at concentrations of 3 + 2 (CI = 2.812) and 4 + 2 (CI = 1.259) μg/mL, and thus could represent an antagonistic effect (Figure 2B). The melittin + cisplatin combinations had CI between 0.7–0.9, indicating a moderate to slight synergism relationship in A2780CR at 2 + 10 (CI = 0.888) and 5 + 10 (CI = 0.741) μg/mL, as shown in Figure 3B. The CI value of melittin + cisplatin was 0.985 at concentrations of 3 + 10 μg/mL and 0.921 at concentrations of 4 + 10 μg/mL used to treat A2780CR, indicating a nearly additive effect. However, the calculated CI was found >1 in A2780CR with the cisplatin+melittin combination at concentrations of 20 + 2 and 30 + 2 μg/mL and thus could represent an antagonism effect. The combination of melittin and cisplatin thus shows potential in the treatment of the resistant cells since there is no evidence of cross resistance against melittin which might be expected as a feature of multidrug resistance. 

### 2.3. Metabolome Analysis

The metabolic effects of melittin in combination with cisplatin on ovarian cancer cells were assessed using an LC-MS based metabolomic approach. Univariate and multivariate statistical analyses were used to examine the effect of combination 1 (5 μg/mL of melittin + 2 μg/mL cisplatin) and combination 2 (2 μg/mL of melittin + 10 μg/mL cisplatin) on A2780 and A2780CR cells, respectively. 

**Figure 4:**
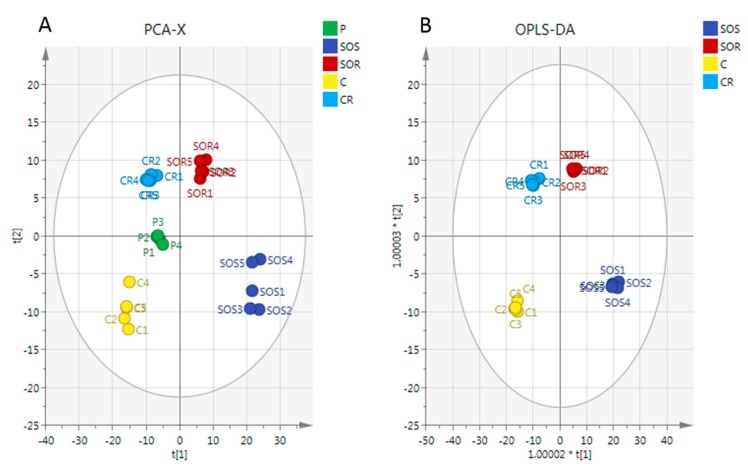
(**A**) Principal components analysis (PCA) vs. (**B**) Orthogonal Partial Least Squares Discriminant Analysis (OPLS-DA). PCA and OPLS-DA scores plot generated from PCA and OPLS-DA using LC-MS normalised data of cells after exposure to the combination (melittin + cisplatin) and controls of A2780 and A2780CR cell lines. A2780-treated cells at 5 μg/mL melittin + 2 μg/mL cisplatin (SOS); untreated A2780 cells (C); A2780CR-treated cells at 2 μg/mL melittin + 10 μg/mL cisplatin (SOR); untreated A2780CR (CR); pooled quality control (QC) samples (P).

A clear separation of A2780 and A2780CR cells was achieved indicating unique metabolite profiles for the treated and control cells on the PCA and OPLS-DA scores plots at both treatment combinations (Figure 4A,B, respectively). The pooled quality control (QC) samples injected at intervals in the analysis run to assess the precision of the measurements and confirm stability of the analytical method produced a single tight cluster in the center of the dataset (Figure 4A), thus validating the analysis. The PCA model parameters and validation of the plot suggested a good model (four components, R2X (cum) = 0.918; Q2 (cum) = 0.856). There was also very clear separations between the treated and control A2780 and A2780CR cells in the OPLS-DA, a supervised model for classifying samples. The OPLS-DA model parameters and validation of the plot suggested a strong model (four components, R2X (cum) = 0.916, R2Y (cum) = 1, Q2 (cum) = 0.975), and the CV-ANOVA for this model was 1.28 × 10^−24^. Hierarchical clustering analysis (HCA) of the metabolomics data showed distinct separation between the control and treated samples (Figure S3). 

**Figure 5:**
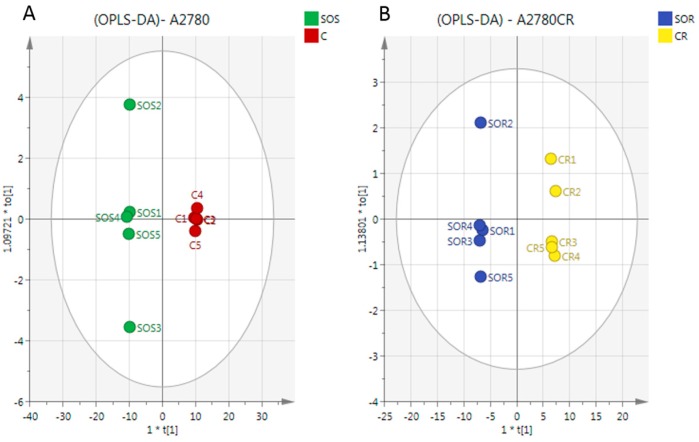
OPLS-DA score plot of (**A**) A2780 cells and (**B**) A2780CR before and after treatment with melittin + cisplatin respectively. Separation of the treated and untreated cells in both cell lines in the model suggests that there are significant metabolite differences induced by treatment in both cell lines. The metabolites responsible for the observed separation are shown in Table 1.

There was a very clear separation of the treated versus untreated A2780 cells obtained by using the OPLS-DA model based on the significant metabolites (Figure 5A). The model parameters and validation of the plot suggested a strong model (two components, R2X (cum) = 0.965, R2Y (cum) = 1, Q2 (cum) = 0.996, CV-ANOVA= 2.62 × 10^−6^). To test the validity, a receiver operator characteristic (ROC) curve and permutation test were also applied (Figure S3). The area under the curve (AUC) for the ROC curve is regarded as excellent when AUC > 0.9. The OPLS-DA model classified the treated and untreated A2780 cells into two groups, and the AUC of the ROC for the groups were in the excellent to perfect classification.

In the case of the A2780CR cells, OPLS-DA models were also generated by comparing control and treated samples based on the significant metabolites (Figure 5B). As in A2780 cells, a clear separation was also found between the treated and untreated A2780CR samples and the CV-ANOVA for this model was 8.42 × 10^−6^ (two components, R2X (cum) = 0.966, R2Y (cum) = 1, Q2 (cum) = 0.994). Furthermore, the validity of the ROC and permutation test showed that the constructed OPLS-DA model was valid (Figures S4 and S5).

For univariate statistical analysis of candidate specific biomarkers in ovarian cancer cells after exposure to the treatment combinations, the false discovery rate statistical test (FDR) was used to reduce the probability of false positive results [23]. Significant changes in the levels of various classes of metabolites, especially those in the mitochondrial TCA cycle, energy metabolism, and nucleotide and amino acids metabolism were observed as summarised in Table 1. The major impression is that the combination of melittin and cisplatin produces distinct metabolic profiles in both cell lines that are unlike those produced when either of the agents are used alone as previously reported [15,24].

It can be observed that there is a strong reduction in the levels of metabolites involved in Krebs cycle and energy metabolism. Several metabolites in the TCA cycle, including citrate, 2-oxoglutarate, malate, and phosphoenolpyruvate were significantly decreased in both sensitive and resistant cells after treatment with the melittin + cisplatin combination. The treatment also reduced the levels of adenosine triphosphate (ATP) in both cell lines. It should be noted that the combination treatment had a stronger effect on ATP levels in the A2780 cells than in the A2780CR cells, although the treatments were not the same as they were based on cell sensitivities towards the two agents. There were also marked decreases in the levels of pentose phosphate pathway metabolites in both cell lines after the combination treatment. Moreover, there were important differences in the levels of purine and pyrimidine metabolites between the two cell lines after treatment with the combinations. With the exception of hypoxanthine and guanine (both purines) which were increased, the rest of these metabolites were significantly decreased by the combination treatment in both cell lines. 

The most affected metabolites due to the combination treatment in both cell lines compared to the other pathways were involved in amino acid metabolism. In A2780 cells, the valine metabolite, 3-methyl-2-oxobutanoic acid, increased, while there were decreases in many amino acids related to arginine and proline metabolism. In A2780CR cells, significant increases were observed in the levels of S-adenosyl-L-methionine, 5′-methylthioadenosine, L-lysine, and L-serine.

## 3. Discussion

Cisplatin is one of the most effective anticancer drugs currently used for treating many types of cancer, however, it comes with serious side effects. Combination therapy has been used in cancer treatment in order to increase therapeutic response and tolerability, and to decrease resistance [2]. 

The present study aimed to determine whether or not melittin, a cytotoxic peptide from bee venom, possesses a synergistic inhibitory effect in combination with cisplatin on A2780 and A2780CR cells. In addition, the study was intended to determine the metabolomics effects of the melittin + cisplatin combination treatment on the two cell lines which would corroborate any observed synergistic cytotoxic effects.

Cell viability assays using the Alamar^®^ Blue method confirmed the synergistic cytotoxic effects of melittin in combination with cisplatin at certain concentrations, although at other concentrations, antagonistic effects were observed. CI analysis of the cytotoxicity data showed that, on A2780 cells, the combinations had synergistic effects at 2 μg/mL of melittin plus either 5 or 6 μg/mL of cisplatin, respectively. In contrast, at melittin + cisplatin concentrations of 3 + 2 and 4 + 2 μg/mL, respectively, antagonistic effects were observed. Synergistic effects were observed in A2780CR cells when the melittin was combined with cisplatin at 2 + 10 and 5 + 10 μg/mL, while with a fixed concentration of melittin (2 μg/mL) and variable concentrations of cisplatin (20 and 30 μg/mL) in the combination, antagonist effects were observed. The CI analysis used the median effect equation of Chou and the combination index equation of Chou and Talalay to quantify synergism or antagonism at different concentrations, and to select the best pair of drugs to combine for potentially maximal antitumor efficacy [22,25]. This method of analysis has been useful in identifying effective combinations of anticancer drugs [26,27].

Recent studies have reported that components of bee venom may exert an anti-tumour effect on human ovarian cancer and that the venom has the potential for enhancing the cytotoxic effect of the antitumor agent cisplatin [14]. Different melittin + cisplatin mechanisms could interact to either reduce or increase anticancer efficacy, thus producing three possible effects: (1) Additive, when the combined effect is equal to the sum of individual effects; (2) Antagonistic, when the effect of one or both compounds is less than when they are applied together than when individually applied; (3) synergism, when the effect of combined substances is greater than the sum of the individual effects [24]. Our findings show that these effects can occur depending on the concentrations of melittin and cisplatin in the combination.

With respect to OCCs, several previous studies have analysed the metabolic responses of OCCs to various compounds [28,29,30]. Additionally, there have been some previous metabolomic studies on the comparison between the effects of cisplatin on squamous cancer cell lines sensitive and resistant to cisplatin [31], and the effects of docetaxel on ovarian cancer stem cells [30]. Our previous study employed a metabolomics approach to assess the effects of melittin monotherapy on OCCs that revealed significant changes in amino acid and carbohydrate metabolism [15]. In addition, clear differences were previously observed in the metabolomes of the untreated cells [15]. Although our study demonstrated profound metabolic changes in the cells after melittin monotherapy, there has been no metabolomics study to date that has comparatively profiled the metabolite composition of OCCs treated with a combination of melittin and cisplatin. A previous study suggested that combination therapy is more effective than monotherapy on cancer cells such as hepatocellular carcinoma [19]. In the current untargeted metabolomics study, metabolic profiles of A2780 and A2780CR cells treated with melittin + cisplatin combinations were assessed using a LC-MS based metabolomics approach, with OPLS-DA models displaying good separation between the experimental groups, high-quality goodness of fit (R^2^), and high-quality goodness of prediction (Q^2^).

The metabolomics analysis demonstrated distinct metabolic profiles for the treated A2780 and A2780CR cells, although the treatments were adjusted in accordance with cell sensitivities. Specifically, the concentrations of melittin and cisplatin used were chosen based on cytotoxicity assays and CI values for synergy to allow detection of a combination effect rather than to achieve a maximal anticancer effect. The most altered metabolites in A2780 and A2780CR cells could be categorised under amino acid, energy, carbohydrate, and nucleotide metabolism. Most of the altered metabolites participate in more than one pathway in significant ways, and the change in that one metabolite could have a resonating effect for other pathways. 

There was a very clear effect of the combination treatment on the purine and pyrimidine pathways. This was very different from the metabolic shifts observed for melittin and cisplatin alone [15,24], suggesting the combination has quite a different effect on cell metabolism. There was a very large increase in the levels of the adenine metabolite, hypoxanthine, and guanine in both cell lines. This possibly indicates that the combination of melittin with cisplatin is promoting greater adduct formation between cisplatin and DNA in comparison with the treatment with cisplatin alone, where there was no strong evidence for effects on the levels of DNA bases [24]. Adducts formed with cisplatin are mainly intra-strand crosslinks joining two guanine residues and to a lesser extent intra-strand links between guanine and adenine [32]. DNA is repaired by excision of damaged bases and this would correlate with largely increased levels of guanine and hypoxanthine, although this presumes that the cisplatin adduct somehow breaks down during the excision. The levels of the purine metabolites adenosine monophosphate (AMP), adenosine diphosphate (ADP), and guanosine triphosphate (GTP) were decreased in both cell lines by the combination treatment. In a previous study, it was found that FK866, a small molecule inhibitor of nicotinamide phosphoribosyltransferase (NAMPT), caused significant metabolic changes in purine metabolism in ovarian cancer and colorectal cancer cells [30]. Moreover, Zhou et al. described a study in hepatocellular carcinoma (HepG2 cells) that showed that high-dose treatment with sorafenib, an oral multikinase inhibitor, affects purine metabolism with significant decreases in GTP levels [33]. Despite the profound dose-dependent metabolic changes in HepG2 cells induced by sorafenib monotherapy, Zhou et al. showed that everolimus, another anticancer agent, in combination with first-line sorafenib therapy results in more pronounced metabolic changes to pyruvate, amino acid, methane, glyoxylate, and dicarboxylate, and glycolysis or gluconeogenesis in hepatocellular carcinoma cells [33]. Other than purine metabolite changes, consistent variations were observed for pyrimidine metabolism. The levels of pyrimidine metabolites such as orotate, dihydrothymine, dihydrouracil, and uridine triphosphate (UTP) were reduced in both cell lines after exposure to the melittin + cisplatin combinations. The reason for the decrease in pyrimidine metabolites is not clear. Normally DNA damage might be associated with increased levels of dihydrothymine which is produced by excision of damaged thymine residues from DNA. 

There were many altered metabolites belonging to several pathways for amino acid metabolism. Most of the metabolites grouped under the arginine and proline pathways were reduced in sensitive cells after the combination treatments; while the arginine metabolite was non-significantly altered in resistant cells. Similarly, our previous study examined the effect of melittin on A2780 and A2780CR cells which showed that the level of arginine was downregulated in cisplatin sensitive cells compared with resistant cells [15]. A number of studies have reported that arginine deficiency enhances apoptosis in different cell lines including human lymphoblastic cell lines [34], mesothelioma cells [35], and melanoma cell lines [36]. Some human cancers, such as melanoma and hepatocellular carcinoma [37], do not express arginosuccinase synthase and therefore are unable to synthesise arginine from citrulline [38]. A recent study observed that ovarian carcinoma SKOV3 cells under arginine deprivation showed increased sensitivity to treatment with paclitaxel, a chemotherapy drug used to treat cancers, at low doses. In this context, it is to be noted that paclitaxel is a disruptor of the cytoskeleton and negatively impacts on the autophagosome-lysosome fusion step [39]. A previous study suggested that combinational treatment based on arginine deprivation and an autophagy inhibitor (for example chloroquine, a known nontoxic antimalarial drug) can potentially be applied as a second line treatment for a subset of ovarian carcinomas deficient in argininosuccinate synthetase [39]. It was also observed that the development of chemoresistance to platinum compounds in ovarian carcinomas leads to collateral appearance of arginine auxotrophy due to the downregulation of argininosuccinate synthetase [40]. The exact mechanism whereby deficiency arginine biosynthesis confers resistance remains unclear.

There was a strong effect of the combination treatment on cellular cysteine and glutathione metabolism; S-glutathionyl-L-cysteine, 3-sulfino-L-alanine, glutathione, L-cysteinylglycine, and L-cystathionine were all lower in cisplatin sensitive cells compared with resistant cells after being treated with the combinations. In a previous study, it was found that the level of glutathione was higher in resistant cells (A2780-CP20) than in sensitive cells (A2780) [41]. This finding resembles the current results in which the level of glutathione was higher in resistant cells than sensitive cells following combination treatment. Two of the main reasons for platinum resistance in OCCs are the p53 mutation and drug-induced increases in intracellular glutathione concentration. A study by Mohell et al. showed that methylene quinuclidinone (MQ), in addition to binding to cysteine residues in p53, also binds to glutathione, decreasing intracellular glutathione levels in OCCs [42]. Therefore, it is possible that the combination promotes greater binding of cisplatin to glutathione thus depleting its levels which occurs to a greater extent in the sensitive cells. Mohell et al. also observed that combination effects of APR-246 (which is a prodrug that is converted to the active compound MQ) with doxorubicin were the cause of a DNA damage response, including activation of the p53 pathway leading to apoptosis [42]. Moreover, recent metabolomics based studies in OCCs have demonstrated that gossypol decreases cellular levels of GSH and induces apoptosis through oxidative stress [30]. In our previous study it was observed that the levels of GSH were no different between resistant and sensitive cells. However, in the current study GSH is depleted by the combination treatment to a much greater extent in the sensitive cells. The depletion appears to be related to the ability of the cells to synthesise GSH. Although there is no difference between the cysteine levels in the two cell lines, there are marked differences in key intermediates which can be used to synthesise both cysteine and GSH including glycine, serine, cystathionine, and glutathione cysteine. There are also lower levels of S-adenosylmethionine in the treated sensitive cells which is a source of homocysteine which is also a precursor of cysteine.

Increased serine biosynthesis is one of many metabolic changes that have been reported in cancer cells [43,44], and serine is a central node for the biosynthesis of many molecules such as glycine and cysteine [45]. High levels of serine in cancer cells have been linked to increased rates of cell proliferation [46]. The level of serine was increased in resistant cells following the combination treatment. In contrast, treatment of the sensitive cells with the combination resulted in a decrease of serine within the cells and a further lowering of the nonessential amino acid glycine. Glycine is incorporated directly into purine nucleotide bases and into GSH. The conversion of serine to glycine, catalysed by serine hydroxymethyltransferase (SHMT), donates a one-carbon unit to tetrahydrofolate to produce 5, 10-methylenetetrahydrofolate (CH_2_-THF). CH_2_-THF is used in thymidine synthesis and is a precursor of other folate species that contribute to purine synthesis [46]. The difference in OCCs could be reflected at the cellular level in terms of differences in the metabolite profiles. Serine is required for a number of biosynthetic and signalling pathways, including the production of phospholipids such as sphingolipids and phosphatidylserine [46]. Previous studies have shown that serine biosynthesis appears to be part of an adaptive response to oxidative stress [47]. The tumour suppressor p53 is emerging as an important regulator of cellular metabolism. P53 is a key player in the cellular response to stress in the form of numerous challenges, including DNA damage, hypoxia, and oncogene activation [48]. Cells lacking p53 fail to respond to serine starvation due to oxidative stress, which leads to reduced viability and severely impaired proliferation [49]. 

The level of ATP was found to be reduced in both cell lines after the combination treatment. ATP was found to be more reduced in both cell lines when treated with the combinations compared with the results observed previously with melittin monotherapy [15]. It is known that glycolysis provides ATP and energy in most cell types, but cancer cells extensively use glycolysis to sustain anabolism, which is necessary for tumour growth [50]. We found that the combinations inhibited glycolysis in both cell lines as indicated by lower levels of fructose bisphosphate and phosphopyruvate. In addition, several TCA cycle intermediates were lowered. Cell death can be executed by different mechanisms, including apoptosis, autophagy, necrosis, or combinations of these processes. Although different cell death mechanisms are unique in their molecular signalling cascades, one molecule is involved in the processes that mediates all types of cell death; ATP. During late-stage apoptosis, ATP levels sharply drop, mostly because of the loss of mitochondrial function and consumption by ATP-dependent proteases. In autophagy, a rescue process of self-degradation to compensate for energy paucity occurs, that also features ATP insufficiency prior to cell death [50,51]. During necrosis, depletion of ATP precedes mitochondrial permeability changes [52]. The fact that ATP deprivation occurs in all types of cell death suggests that energy metabolism may play a critical role in the survival of cancer cells under stress. Thus, it could be possible that the A2780 cells may be undergoing late-stage apoptosis cell death in response to the combination treatment whereas the A2780CR may be undergoing early-stage apoptosis cell death. In our study, we found that combination treatment probably inhibited glycolysis in A2780 cells by depletion of NAD^+^. Moreover, the level of NAD^+^ was found to be decreased in A2780CR cells after combination treatment. It appears that the combinations had more impact on the oxidative phosphorylation pathway in both cell lines in comparison with melittin as a single treatment [15]. The inhibition of NAMPT leads to suppression of tumour cell growth and induction of apoptosis due to NAD^+^ depletion [53]. NAMPT represents a promising therapeutic target for the development of potential novel cancer drugs [54,55]. In most cancer cells, poly (ADP-ribose) polymerase is activated due to DNA damage and cell death induced by oxidative stress [56,57]. Therefore, NAMPT inhibition leads to attenuation of glycolysis, resulting in further alteration of the carbohydrate metabolism in the cells [53]. 

## 4. Materials and Methods 

### 4.1. Cell Lines and Cultures

The cisplatin-sensitive (A2780) and resistant (A2780CR) human ovarian carcinoma cells were obtained from ECACC (Porton Down, Salisbury, UK) and maintained at 75 × 10^4^ cells/mL in RPMI 1640 medium (Lonza, Verviers, Belgium) supplemented with 1% (v/v) l-glutamine (Invitrogen, Paisley, UK), 100 IU/mL/100 μg/mL penicillin/streptomycin (Invitrogen, Paisley, UK), and 10% (v/v) foetal bovine serum (FBS) (Life Technologies, Carlsbad, CA, USA). In addition, the cultures for the A2780CR cells contained 3 μg/mL of cisplatinum (Tocris Bioscience, Bristol, UK) in the first three passages. Sub-confluent cultures were split by trypsinisation every 4–5 days and maintained at 37 °C in a humidified atmosphere saturated with 5% CO_2_. 

### 4.2. Cell Viability Assay

Cisplatin was purchased from Tocris Bioscience (Bristol, UK) and was dissolved in sterile water with gentle warming according to the manufacturer’s instructions. Melittin was purified from bee venom (supplied by Beesen Co. Ltd., Dae Jeon, Korea) by reversed phase liquid chromatography [58] and reconstituted in sterile water to form a stock solution of 1 mg/mL before storage at −20 °C until required. Cell viability was assessed by an Alamar^®^ Blue cell assay (Thermo Fisher Scientific, Loughborough, UK), as previously described [15]. 

To test the synergistic cytotoxic effect, experiments were performed with A2780 and A2780CR cell lines using various combinations of melittin with cisplatin in medium, to assess possible synergistic/additive effects. Both A2780 and A2780CR cells were seeded at 1 × 10^4^ cells/well in 96-well plates (Corning^®^, Sigma-Aldrich, Poole, UK) and incubated at 37 °C and 5% CO_2_ in a humidified atmosphere for 24 h. For the A2780 cell line, 3, 4, 5, and 6 μg/mL of melittin was combined with 2 μg/mL of cisplatin and 3, 4, and 5 μg/mL of cisplatin was combined with 3 μg/mL of melittin. For the A2780CR cell line, 2, 3, 4 and 5 μg/mL of melittin was combined with 10 μg/mL of cisplatin and 20 and 30 μg/mL of cisplatin was combined with 2 μg/mL of melittin. Twenty hours after drug treatment, AB was added at a final concentration of 10% (v/v) and the resultant mixture was incubated for a further 4 h at 37 °C and 5% CO_2_. Then, the plates were read at an excitation wavelength of 560 nm and the emission at 590 nm was recorded on a SpectraMax M3 microplate reader (Molecular Devices, Sunnyvale, CA, USA). All experiments were performed in triplicate.

### 4.3. Calculation of CI

The specific interaction between melittin and cisplatin on A2780 and A2780CR cancer cell lines was evaluated by the CI analysis. Drug combination synergy was performed using CompuSyn software [59]. CI values and CI-Fa plot (plot representing CI versus Fa, the fraction affected by a particular dose) were calculated by CompuSyn program (Compusyn Inc., Paramus, NJ, USA). All experiments were repeated at least three times. 

### 4.4. Statistical Analysis

GraphPad Prism for Windows (version 5.00, GraphPad Software, San Diego, CA, USA) was employed to produce dose-response curves by performing nonlinear regression analysis of the cell viability data. The mean IC_50_ values were calculated from at least three measurements of independent experiments (*n* = 3). 

### 4.5. Determination of the Effect of Melittin in Combination with Cisplatin on Cell Metabolomes

The A2780 cell line was treated with the combination of melittin and cisplatin at concentrations of 5 and 2 μg/mL, respectively, for 24 h (*n* = 5). The A2780CR cells were treated with the combination of melittin and cisplatin at concentrations of 2 and 10 μg/mL, respectively, for 24 h (*n* = 5). The cells were seeded at 75 × 10^4^ cells/mL in T-25 cell culture flasks and incubated for 1 doubling time (48 h) before treatment with the combinations and incubation for an additional 24 h. After the treatment, the medium was removed and the cells were washed twice with 3 mL of phosphate-buffered saline (PBS) at 37 °C before lysis. Cell lysates were prepared by extraction with ice cold methanol:acetonitrile:water (50:30:20) (1 mL per 2 × 10^6^ cells). The cells were scraped and cell lysates mixed on a Thermo mixer at 1440 rotations per minute (r.p.m.) for 12 min at 4 °C, before being centrifuged at 13500 r.p.m. for 15 min at 0 °C. The supernatants were collected and transferred into HPLC vials for LC-MS analysis. During the analysis, the temperature of the autosampler was maintained at 4 °C. Mixtures of standard metabolites (Sigma-Aldrich, Poole, UK) and the pooled quality control (QC) sample were injected in each analysis run in order to facilitate identification and to evaluate the stability and reproducibility of the analytical method. The pooled QC sample was obtained by taking equal aliquots from all the samples and placing them into the same HPLC vial.

### 4.6. LC-MS Conditions 

Liquid chromatographic separation was carried out on an Accela HPLC system interfaced to an Exactive Orbitrap mass spectrometer (Thermo Fisher Scientific, Bremen, Germany) using a hydrophilic interaction liquid chromatography (HILIC) column (ZIC-pHILIC, 150 × 4.6 mm, 5 μm particle size) supplied by Hichrom Ltd. (Reading, UK). The method was reported previously [60]. Briefly, the mobile phase for ZIC-pHILIC consisted of 20 mM ammonium carbonate (Sigma-Aldrich, Poole, UK) in water purified by Direct-Q3 Ultrapure water purification system (Millipore, UK) at pH 9.2 (solvent A) and acetonitrile (Sigma-Aldrich, Poole, UK) (solvent B) at a flow rate of 0.3 mL/min. The elution gradient was an A:B ratio of 20:80 at 0 min, 80:20 at 30 min, 92:8 at 35 min, and finally 20:80 at 45 min. 

### 4.7. Data Extraction and Analysis

Data extraction for each of the samples was carried out by MZmine-2.10 software (mzmine.github.io/) using identical parameters for peak detection, deconvolution, deisotoping, alignment, filtering, and gap filling in order to make multiple data files comparable [23]. The extracted ions, with their corresponding m/z values and retention times, were pasted into an Excel macro of the most common metabolites prepared in–house to facilitate identification, and a library search was also carried out against accurate mass data of the metabolites in the Human Metabolome, Kyoto Encyclopedia of Genes and Genomes, and Metlin databases. The lists of the metabolites obtained from these searches were then carefully evaluated manually by considering the quality of their peaks and their retention time match to the standard metabolite mixtures run in the same sequence. The MS data were log_2_-transformed and mean-centred with unit variance scaling for statistical analysis. Statistical analyses were performed using both univariate and multivariate approaches. The *p*-values from univariate analysis were adjusted using the FDR control and differences in the levels (or peak areas) of the metabolites between treated and control cells were considered significant at *p* < 0.05. MetaboAnalyst^®^, a web-based metabolomic data processing tool [60], was used for supporting fold-change analysis, and *t*-tests. SIMCA-P software version 14.0 (Umetrics, Crewe, UK) was also used for multivariate analysis of the metabolite data with Pareto scaling prior to modelling with PCA and OPLS-DA. OPLS-DA models were validated based on multiple correlation coefficient (*R*^2^) and cross-validated R^2^ (*Q*^2^) in cross-validation and permutation tests.

## 5. Conclusions

Based on the results presented, a metabolic signature for the cisplatin and melittin combination treatment for A2780 and A2780CR OCCs is proposed. Melittin and cisplatin together have different metabolic effects on these cells compared to melittin alone, which preferentially affects fatty acids, amino acids, and TCA cycle intermediates. The most significantly affected metabolites due to the melittin + cisplatin combination treatment in both cell lines were in the TCA cycle, oxidative phosphorylation, purine and pyrimidine metabolism, and arginine/proline pathways. This distinct mechanism of action of the melittin + cisplatin combination may provide a new paradigm for overcoming chemoresistance in ovarian cancer therapy. Our results provide rationale for the ongoing study of melittin in combination with cisplatin that could produce improved therapy for platinum resistance in ovarian cancer.

## Figures and Tables

**Table 1:** Metabolites significantly altered by treatment with melittin in combination with cisplatin at 5 μg/mL melittin + 2 μg/mL cisplatin on A2780 and at 2 μg/mL melittin + 10 μg/mL cisplatin on A2780CR.

m/z	RT (min)	Metabolites	SR/CR		SS/C	
			*p*-Value	Ratio	*p*-Value	Ratio
**Citrate cycle (TCA cycle)/glycolysis**						
338.989	18.1	* D-Fructose 1,6-bisphosphate	<0.001	0.278	<0.001	0.246
115.004	16.0	* Fumarate	<0.001	0.227	<0.01	0.402
133.014	16.1	* (S)-Malate	<0.001	0.218	<0.001	0.062
145.014	15.7	* 2-Oxoglutarate	<0.001	0.324	<0.001	0.048
191.02	18.1	* Citrate	<0.001	0.588	<0.001	0.172
173.009	17.9	* cis-Aconitate	<0.001	0.490	<0.001	0.072
166.975	17.5	* Phosphoenolpyruvate	<0.001	0.344	<0.05	0.082
**Oxidative phosphorylation**						
664.116	14.2	* NAD+	<0.001	0.294	<0.001	0.067
508.003	16.2	* ATP	<0.001	0.362	<0.001	0.073
**Glycine/Serine/Cysteine and Glutathione**						
241.031	16.3	* L-Cystine	ns	1.739	ns	1.832
427.095	17.1	S-Glutathionyl-L-cysteine	ns	0.750	<0.001	0.218
152.002	14.6	3-Sulfino-L-alanine	ns	0.706	<0.001	0.139
308.091	14.5	* Glutathione (GSH)	<0.001	0.562	<0.001	0.092
179.048	14.4	L- Cysteinylglycine (Cys-Gly)	<0.001	0.521	<0.001	0.067
223.074	17.1	* L-Cystathionine	<0.001	0.438	<0.001	0.004
76.0394	15.7	* Glycine	ns	1.050	<0.001	0.142
116.035	10.8	L-2-Amino-3-oxobutanoic acid	<0.001	0.319	<0.001	0.317
106.050	15.8	* L-Serine	<0.001	7.112	<0.001	0.420
**Pentose phosphate pathway**						
195.051	13.7	* D-Gluconic acid	<0.001	0.147	<0.001	0.093
308.978	16.5	D-Ribose 1,5-bisphosphate	<0.001	0.259	<0.001	0.124
149.046	11.9	* D-Ribose	<0.001	0.222	<0.001	0.285
229.012	15.8	* D-Ribose 5-phosphate	<0.001	0.545	<0.001	0.131
**Lysine biosynthesis**						
170.046	14.3	2,3,4,5-Tetrahydrodipicolinate	<0.001	0.245	<0.001	0.112
147.113	24.3	* L-Lysine	<0.001	1.966	ns	0.724
162.112	13.3	* L-Carnitine	<0.001	0.508	<0.001	0.135
243.074	17.2	5-Phosphonooxy-L-lysine	<0.001	0.303	<0.001	0.006
128.071	15.5	2,3,4,5-Tetrahydropyridine-2-carboxylate	<0.001	0.433	<0.001	0.271
**Purine metabolism**						
137.046	10.2	* Hypoxanthine	<0.001	16.99	<0.01	7.87
152.056	12.4	* Guanine	<0.001	13.19	<0.01	102.21
348.07	13.8	* AMP	<0.001	0.372	<0.001	0.190
428.036	15.0	* ADP	<0.001	0.376	<0.001	0.163
442.018	17.8	GDP	<0.001	0.509	ns	0.104
521.984	18.9	* GTP	<0.001	0.442	<0.001	0.079
426.013	16.9	Adenylyl sulfate	<0.001	0.271	<0.01	0.014
**Pyrimidine metabolism**						
155.01	10.3	* Orotate	<0.001	0.218	<0.001	0.050
129.066	14.8	5,6-Dihydrothymine	<0.001	0.326	<0.001	0.120
480.982	15.8	dTTP	<0.001	0.337	<0.001	0.347
175.036	16.8	N-Carbamoyl-L-aspartate	<0.001	0.036	<0.001	0.017
115.05	14.7	5,6-Dihydrouracil	<0.001	0.383	<0.001	0.079
402.995	16.4	* UDP	<0.001	0.485	<0.001	0.035
484.975	17.6	* UTP	<0.001	0.465	<0.001	0.059
323.029	15.2	* UMP	ns	1.195	<0.001	0.282
**Arginine/Proline/Glutamate/Methionine **						
188.057	14.3	N-Acetyl-L-glutamate	<0.001	0.241	<0.001	0.029
176.103	15.8	* L-Citrulline	<0.001	0.521	<0.001	0.189
173.104	25.8	* L-Arginine	ns	1.161	<0.001	0.379
130.051	14.5	L-Glutamate-5-semialdehyde	<0.001	0.538	<0.001	0.235
116.071	12.8	* L-Proline	<0.001	0.568	<0.001	0.228
399.144	16.3	* S-Adenosyl-L-methionine	<0.001	2.005	<0.001	0.115
298.096	6.4	* 5′-Methylthioadenosine	<0.05	2.016	<0.001	0.158
146.093	15.1	* 4-Guanidinobutanoate	<0.001	0.325	<0.001	0.055
291.129	16.8	N-(L-Arginino) succinate	<0.001	0.260	<0.001	0.010
247.14	14.2	N_2_-(D-1-Carboxyethyl)-L-arginine	<0.001	0.170	<0.001	0.010
174.087	15.3	5-Guanidino-2 oxopentanoate	<0.001	0.680	<0.001	0.058
132.077	14.7	* Creatine	<0.001	0.403	<0.001	0.075
210.029	15.2	* Phosphocreatine	<0.001	0.422	<0.001	0.051
**Miscellaneous**						
110.027	14.9	Hypotaurine	<0.001	0.139	<0.001	0.009
115.04	8.2	3-Methyl-2-oxobutanoic acid	<0.001	0.191	<0.05	5.604
166.053	13.4	L-Methionine S-oxide	<0.01	0.470	<0.001	0.250
218.067	13.9	O-Succinyl-L-homoserine	<0.001	0.082	<0.001	0.011
181.051	9.0	3-(4-Hydroxyphenyl)lactate	<0.001	0.656	<0.001	0.087
204.123	11.1	* O-Acetylcarnitine	<0.001	0.137	<0.001	0.021
176.056	10.3	4-Hydroxy-4-methylglutamate	ns	1.911	<0.001	0.022
159.076	15.8	4-Methylene-L-glutamine	<0.001	0.607	ns	0.258
175.025	14.4	* Ascorbate	<0.001	0.067	<0.001	0.024
165.041	13.0	L-Arabinonate	<0.001	0.372	<0.001	0.166
179.056	17.1	Hexose	<0.05	0.670	<0.05	0.334

RT: min; SR: combination 2 treated A2780CR; CR: control A2780CR; SS: combination 1 treated A2780; C: control A2780; ns: non-significant. * Retention time matches standard.

## Supplementary Materials

The following are available online at www.mdpi.com/2218-1989/7/2/14/s1, Figure S1: Cell viability was determined following treatment with cisplatin for 24 h (A) IC_50_ = 10.8 µg/mL A2780CR; (B) IC_50_ = 4.9 µg/mL A2780, Figure S2: Cell viability was determined following treatment with melittin for 24 h (IC_50_ = 6.8 µg/mL A2780; IC_50_ = 4.5 µg/mL A2780CR), Figure S3: Hierarchical clustering analysis (HCA) of 20 ovarian cancer cell samples. It shows two main groups and four subgroups. The groups: CR, control of cisplatin resistance cell lines; SOR: A2780CR after treatment with melittin + cisplatin; C, control of cisplatin sensitive cell lines; SOS, A2780 after treatment with melittin + cisplatin, Figure S4: (A) Permutation analysis of OPLS-DA model derived from A2780 cells treated with melittin/cisplatin and controls cells. Statistical validation of the OPLS-DA model by permutation analysis using 100 different model permutations. The goodness of fit (R2) and predictive capability (Q2) of the original model are indicated on the far right and remain higher than those of the 100 permuted models to the left. OPLS-DA, orthogonal partial least squares discriminant analysis. (B) Receiver Operating Characteristics (ROC) curve shows sensitivity (true positive rate (TPR)) on the y-axis versus (false positive rate (FPR = 1 - Specificity)) on the x-axis. The area under ROC curve (AUROCC) = 1 for SOS and C groups, Figure S5: (A) Permutation analysis of OPLS-DA model derived from A2780CR cells treated with melittin/cisplatin and controls cells. Statistical validation of the OPLS-DA model by permutation analysis using 100 different model permutations. The goodness of fit (R2) and predictive capability (Q2) of the original model are indicated on the far right and remain higher than those of the 100 permuted models to the left. OPLS-DA, orthogonal partial least squares discriminant analysis. (B) Receiver Operating Characteristics (ROC) curve shows sensitivity (true positive rate (TPR)) on the y-axis versus (false positive rate (FPR = 1 - Specificity)) on the x-axis. The area under ROC curve (AUROCC) = 1 for SOR and CR groups.

## References

[1] Takakura M., Nakamura M., Kyo S., Hashimoto M., Mori N., Ikoma T., Mizumoto Y., Fujiwara T., Urata Y., Inoue M. (2010). Intraperitoneal administration of telomerase-specific oncolytic adenovirus sensitizes ovarian cancer cells to cisplatin and affects survival in a xenograft model with peritoneal dissemination. Cancer Gene Ther..

[2] Pinto A.C., Moreira J.N., Simões S. (2011). Combination Chemotherapy in Cancer: Principles, Evaluation and Drug Delivery Strategies.

[3] Fontaine F., Overman J., François M. (2015). Pharmacological manipulation of transcription factor protein-protein interactions: Opportunities and obstacles. Cell Regen..

[4] Kim H., Lee G., Park S., Chung H.-S., Lee H., Kim J.-Y., Nam S., Kim S.K., Bae H. (2013). Bee venom mitigates cisplatin-induced nephrotoxicity by regulating CD4. Evid.-Based Complement. Altern. Med..

[5] Kim H., Lee H., Lee G., Jang H., Kim S.-S., Yoon H., Kang G.-H., Hwang D.-S., Kim S.K., Chung H.-S. (2015). Phospholipase A_2_ inhibits cisplatin-induced acute kidney injury by modulating regulatory T cells by the CD206 mannose receptor. Kidney Int..

[6] Chvetzoff G., Bonnotte B., Chauffert B. (1998). Anticancer chemotherapy. Prevention of toxicity. Presse Med..

[7] Choi K.E., Hwang C.J., Gu S.M., Park M.H., Kim J.H., Park J.H., Ahn Y.J., Kim J.Y., Song M.J., Song H.S. (2014). Cancer cell growth inhibitory effect of bee venom via increase of death receptor 3 expression and inactivation of NF-kappa B in NSCLC cells. Toxins.

[8] Lim B.-S., Moon H.J., Li D.X., Gil M., Min J.K., Lee G., Bae H., Kim S.K., Min B.-I. (2013). Effect of bee venom acupuncture on oxaliplatin-induced cold allodynia in rats. Evid.-Based Complement. Altern. Med..

[9] Yoon J., Jeon J.-H., Lee Y.-W., Cho C.-K., Kwon K.-R., Shin J.-E., Sagar S., Wong R., Yoo H.-S. (2012). Sweet bee venom pharmacopuncture for chemotherapy-induced peripheral neuropathy. J. Acupunct. Meridian Stud..

[10] Gajski G., Čimbora-Zovko T., Rak S., Osmak M., Garaj-Vrhovac V. (2016). Antitumour action on human glioblastoma A1235 cells through cooperation of bee venom and cisplatin. Cytotechnology.

[11] Gajski G., Čimbora-Zovko T., Rak S., Rožman M., Osmak M., Garaj-Vrhovac V. (2014). Combined antitumor effects of bee venom and cisplatin on human cervical and laryngeal carcinoma cells and their drug resistant sublines. J. Appl. Toxicol..

[12] Lee G., Bae H. (2016). Bee venom phospholipase A_2_: Yesterday’s enemy becomes today’s friend. Toxins.

[13] Kinsey G.R., Okusa M.D. (2014). Expanding role of T cells in acute kidney injury. Curr. Opin. Nephrol. Hypertens..

[14] Alizadehnohi M., Nabiuni M., Nazari Z., Safaeinejad Z., Irian S. (2012). The synergistic cytotoxic effect of cisplatin and honey bee venom on human ovarian cancer cell line A2780cp. J. Venom Res..

[15] Alonezi S., Tusiimire J., Wallace J., Dufton M.J., Parkinson J.A., Young L.C., Clements C.J., Park J.K., Jeon J.W., Ferro V.A. (2016). Metabolomic profiling of the effects of melittin on cisplatin resistant and cisplatin sensitive ovarian cancer cells using mass spectrometry and Biolog microarray technology. Metabolites.

[16] Palmnas M.S., Vogel H.J. (2013). The future of NMR metabolomics in cancer therapy: Towards personalizing treatment and developing targeted drugs?. Metabolites.

[17] Spratlin J.L., Serkova N.J., Eckhardt S.G. (2009). Clinical applications of metabolomics in oncology: A review. Clin. Cancer Res..

[18] Odunsi K., Wollman R.M., Ambrosone C.B., Hutson A., McCann S.E., Tammela J., Geisler J.P., Miller G., Sellers T., Cliby W. (2005). Detection of epithelial ovarian cancer using ^1^H-NMR-based metabonomics. Int. J. Cancer.

[19] Zheng J.-F., Lu J., Wang X.-Z., Guo W.-H., Zhang J.-X. (2015). Comparative metabolomic profiling of hepatocellular carcinoma cells treated with sorafenib monotherapy vs. sorafenib-everolimus combination therapy. Med. Sci. Monit. Int. Med. J. Exp. Clin. Res..

[20] Patrick Reynolds C., Maurer B.J. (2005). Evaluating response to antineoplastic drug combinations in tissue culture models. Chemosensitivity.

[21] Chou T.-C., Talalay P. (1983). Analysis of combined drug effects: A new look at a very old problem. Trends Pharmacol. Sci..

[22] Chou T.-C., Talalay P. (1984). Quantitative analysis of dose-effect relationships: The combined effects of multiple drugs or enzyme inhibitors. Adv. Enzyme Regul..

[23] Xia J., Wishart D.S. (2016). Using MetaboAnalyst 3.0 for Comprehensive Metabolomics Data Analysis. Curr. Protoc. Bioinform..

[24] Alonezi S., Al Washih M., Clements C.J., Young L., Ferro V.A., Watson D.G. (2017). Current liquid chromatography mass spectrometry (LCMS) and phenotype microarray profiling of ovarian cancer cells after exposure to cisplatin. Curr. Metabol..

[25] Chou T.-C., Motzer R.J., Tong Y., Bosl G.J. (1994). Computerized quantitation of synergism and antagonism of taxol, topotecan, and cisplatin against human teratocarcinoma cell growth: A rational approach to clinical protocol design. J. Natl. Cancer Inst..

[26] Chang T., Gulati S., Chou T., Vega R., Gandola L., Ibrahim S.E., Yopp J., Colvin M., Clarkson B. (1985). Synergistic effect of 4-hydroperoxycyclophosphamide and etoposide on a human promyelocytic leukemia cell line (HL-60) demonstrated by computer analysis. Cancer Res..

[27] Bible K.C., Kaufmann S.H. (1997). Cytotoxic synergy between flavopiridol (NSC 649890, L86-8275) and various antineoplastic agents: The importance of sequence of administration. Cancer Res..

[28] Wang J., Jin L., Li X., Deng H., Chen Y., Lian Q., Ge R., Deng H. (2013). Gossypol induces apoptosis in ovarian cancer cells through oxidative stress. Mol. Biosyst..

[29] Vermeersch K.A., Wang L., McDonald J.F., Styczynski M.P. (2014). Distinct metabolic responses of an ovarian cancer stem cell line. BMC Syst. Biol..

[30] Tolstikov V., Nikolayev A., Dong S., Zhao G., Kuo M.-S. (2014). Metabolomics analysis of metabolic effects of nicotinamide phosphoribosyltransferase (NAMPT) inhibition on human cancer cells. PLoS ONE.

[31] Huang Y., Bell L.N., Okamura J., Kim M.S., Mohney R.P., Guerrero-Preston R., Ratovitski E.A. (2012). Phospho-ΔNp63α/SREBF1 protein interactions: Bridging cell metabolism and cisplatin chemoresistance. Cell Cycle.

[32] Chaney S.G., Campbell S.L., Bassett E., Wu Y. (2005). Recognition and processing of cisplatin- and oxaliplatin-DNA adducts. Crit. Rev. Oncol./Hematol..

[33] Zhou S., Luo R. (2013). Metabolomic response to sorafenib treatment in human hepatocellular carcinoma cells. FASEB J..

[34] Gong H., Zölzer F., Von Recklinghausen G., Havers W., Schweigerer L. (2000). Arginine deiminase inhibits proliferation of human leukemia cells more potently than asparaginase by inducing cell cycle arrest and apoptosis. Leukemia.

[35] Szlosarek P.W., Klabatsa A., Pallaska A., Sheaff M., Smith P., Crook T., Grimshaw M.J., Steele J.P., Rudd R.M., Balkwill F.R. (2006). In vivo loss of expression of argininosuccinate synthetase in malignant pleural mesothelioma is a biomarker for susceptibility to arginine depletion. Clin. Cancer Res..

[36] Feun L., You M., Wu C., Kuo M., Wangpaichitr M., Spector S., Savaraj N. (2008). Arginine deprivation as a targeted therapy for cancer. Curr. Pharm. Des..

[37] Ensor C.M., Holtsberg F.W., Bomalaski J.S., Clark M.A. (2002). Pegylated arginine deiminase (ADI-SS PEG_20, 000 mw_) inhibits human melanomas and hepatocellular carcinomas in vitro and in vivo. Cancer Res..

[38] Lind D.S. (2004). Arginine and cancer. J. Nutr..

[39] Shuvayeva G., Bobak Y., Igumentseva N., Titone R., Morani F., Stasyk O., Isidoro C. (2014). Single amino acid arginine deprivation triggers prosurvival autophagic response in ovarian carcinoma SKOV3. BioMed Res. Int..

[40] Nicholson L.J., Smith P.R., Hiller L., Szlosarek P.W., Kimberley C., Sehouli J., Koensgen D., Mustea A., Schmid P., Crook T. (2009). Epigenetic silencing of argininosuccinate synthetase confers resistance to platinum-induced cell death but collateral sensitivity to arginine auxotrophy in ovarian cancer. Int. J. Cancer.

[41] Poisson L.M., Munkarah A., Madi H., Datta I., Hensley-Alford S., Tebbe C., Buekers T., Giri S., Rattan R. (2015). A metabolomic approach to identifying platinum resistance in ovarian cancer. J. Ovarian Res..

[42] Mohell N., Alfredsson J., Fransson Å., Uustalu M., Byström S., Gullbo J., Hallberg A., Bykov V., Björklund U., Wiman K. (2015). Apr-246 overcomes resistance to cisplatin and doxorubicin in ovarian cancer cells. Cell Death Dis..

[43] Davis J.L., Fallon H.J., Morris H.P. (1970). Two enzymes of serine metabolism in rat liver and hepatomas. Cancer Res..

[44] Snell K. (1984). Enzymes of serine metabolism in normal, developing and neoplastic rat tissues. Adv. Enzyme Regul..

[45] Locasale J.W. (2013). Serine, glycine and one-carbon units: Cancer metabolism in full circle. Nat. Rev. Cancer.

[46] Mattaini K.R., Sullivan M.R., Vander Heiden M.G. (2016). The importance of serine metabolism in cancer. J. Cell Biol..

[47] Maddocks O.D., Berkers C.R., Mason S.M., Zheng L., Blyth K., Gottlieb E., Vousden K.H. (2013). Serine starvation induces stress and p53-dependent metabolic remodelling in cancer cells. Nature.

[48] Vousden K.H., Prives C. (2009). Blinded by the light: The growing complexity of p53. Cell.

[49] Amelio I., Cutruzzolá F., Antonov A., Agostini M., Melino G. (2014). Serine and glycine metabolism in cancer. Trends Biochem. Sci..

[50] Lemasters J.J., Qian T., He L., Kim J.-S., Elmore S.P., Cascio W.E., Brenner D.A. (2002). Role of mitochondrial inner membrane permeabilization in necrotic cell death, apoptosis, and autophagy. Antioxid. Redox Signal..

[51] Skulachev V. (2006). Bioenergetic aspects of apoptosis, necrosis and mitoptosis. Apoptosis.

[52] Vanlangenakker N., Berghe T.V., Krysko D.V., Festjens N., Vandenabeele P. (2008). Molecular mechanisms and pathophysiology of necrotic cell death. Curr. Mol. Med..

[53] Tan B., Dong S., Shepard R.L., Kays L., Roth K.D., Geeganage S., Kuo M.-S., Zhao G. (2015). Inhibition of nicotinamide phosphoribosyltransferase (NAMPT), an enzyme essential for NAD^+^ biosynthesis, leads to altered carbohydrate metabolism in cancer cells. J. Biol. Chem..

[54] Giannetti A.M., Zheng X., Skelton N.J., Wang W., Bravo B.J., Bair K.W., Baumeister T., Cheng E., Crocker L., Feng Y. (2014). Fragment-based identification of amides derived from trans-2-(pyridin-3-yl) cyclopropanecarboxylic acid as potent inhibitors of human nicotinamide phosphoribosyltransferase (NAMPT). J. Biol. Chem..

[55] Zheng X., Bauer P., Baumeister T., Buckmelter A.J., Caligiuri M., Clodfelter K.H., Han B., Ho Y.-C., Kley N., Lin J. (2013). Structure-based discovery of novel amide-containing nicotinamide phosphoribosyltransferase (NAMPT) inhibitors. J. Biol. Chem..

[56] Yu S.-W., Wang H., Poitras M.F., Coombs C., Bowers W.J., Federoff H.J., Poirier G.G., Dawson T.M., Dawson V.L. (2002). Mediation of poly (ADP-ribose) polymerase-1-dependent cell death by apoptosis-inducing factor. Science.

[57] Du L., Zhang X., Han Y.Y., Burke N.A., Kochanek P.M., Watkins S.C., Graham S.H., Carcillo J.A., Szabó C., Clark R.S. (2003). Intra-mitochondrial poly (ADP-ribosylation) contributes to NAD^+^ depletion and cell death induced by oxidative stress. J. Biol. Chem..

[58] Tusiimire J., Wallace J., Dufton M., Parkinson J., Clements C.J., Young L., Park J.K., Jeon J.W., Watson D.G. (2015). An LCMS method for the assay of melittin in cosmetic formulations containing bee venom. Anal. Bioanal. Chem..

[59] Chou T., Martin N. (2005). Compusyn for Drug Combinations: Pc Software and User’s Guide: A Computer Program for Quantitation of Synergism and Antagonism in Drug Combinations, and the Determination of IC_50_ and ED_50_ and LD_50_ Values.

[60] Zhang R., Watson D.G., Wang L., Westrop G.D., Coombs G.H., Zhang T. (2014). Evaluation of mobile phase characteristics on three zwitterionic columns in hydrophilic interaction liquid chromatography mode for liquid chromatography-high resolution mass spectrometry based untargeted metabolite profiling of leishmania parasites. J. Chromatogr. A.

