# Supplementary material for: Metabolomic Profiling of the Synergistic Effects of Melittin in Combination with Cisplatin on Ovarian Cancer Cells
Source: Metabolites. 2017 Apr 14;7(2):14. doi: 10.3390/metabo7020014 (PMC5487985; doi:10.3390/metabo7020014)
Supplement: Supplementary file 1 [file metabolites-07-00014-s001.pdf]

Supplementary Materials: Metabolomic Profiling of the Synergistic Effects of Melittin in Combination with Cisplatin on Ovarian Cancer Cells

Sanad Alonezi, Jonans Tusiimire, Jennifer Wallace, Mark J. Dufton, John A. Parkinson, Louise C. Young, Carol J. Clements, Jin-Kyu Park, Jong-Woon Jeon, Valerie A. Ferro and David G. Watson

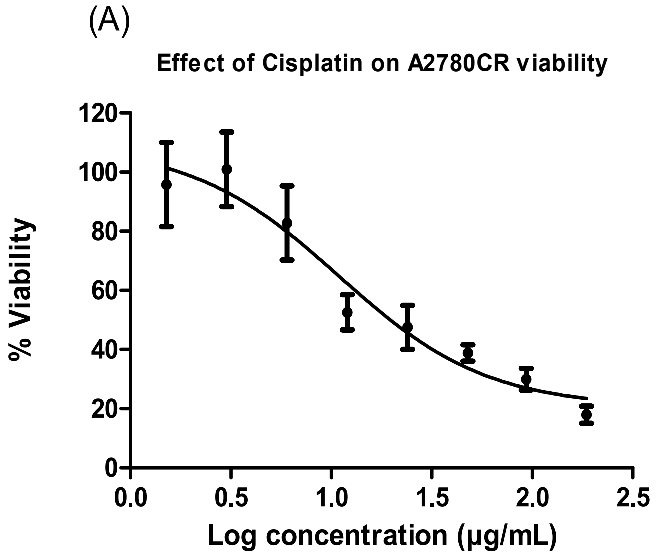

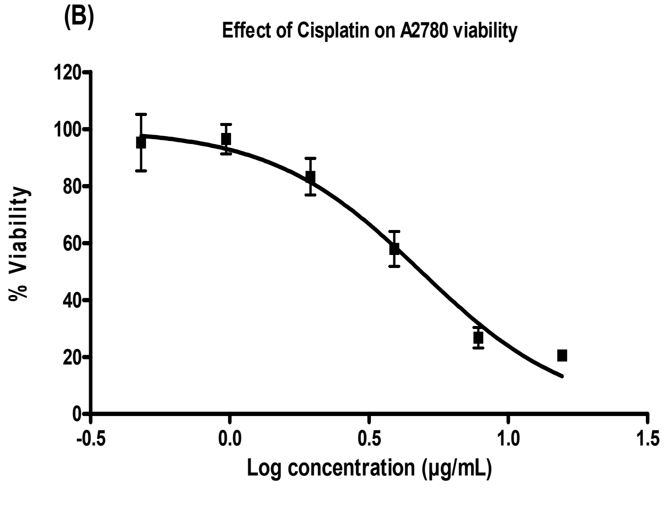

Figure S1. Cell viability was determined following treatment with cisplatin for 24 h (A) IC_{50} = 10.8 µg/mL A2780CR; (B) IC_{50} = 4.9 µg/mL A2780.

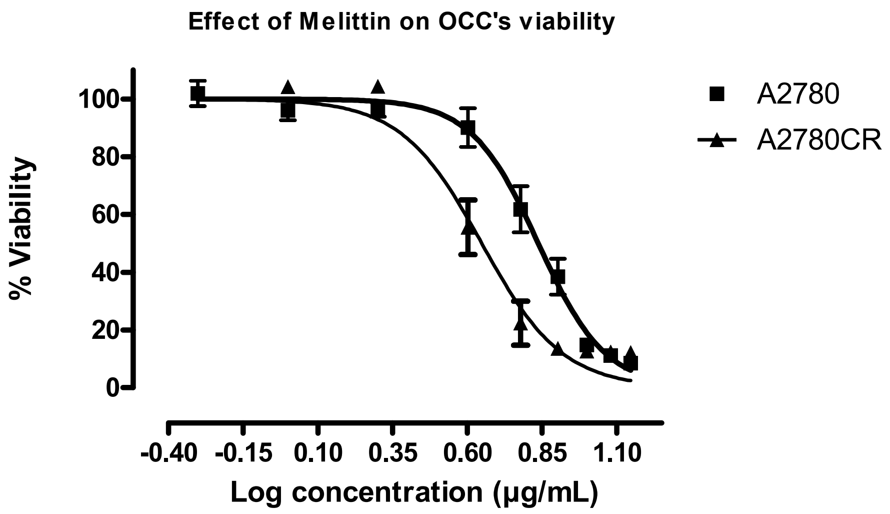

Figure S2. Cell viability was determined following treatment with melittin for 24 h (IC_{50} = 6.8μg/mL A2780; IC_{50} = 4.5μg/mL A2780CR).

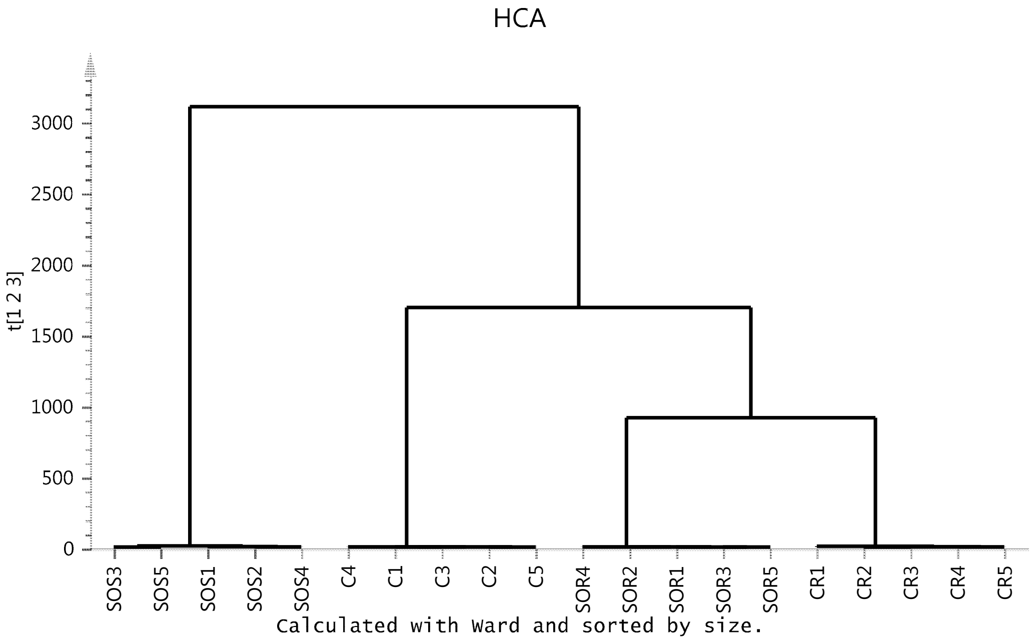

Figure S3. Hierarchical clustering analysis (HCA) of 20 ovarian cancer cell samples. It shows two main groups and four subgroups. The groups: CR, control of cisplatin resistance cell lines; SOR: A2780CR after treatment with melittin + cisplatin; C, control of cisplatin sensitive cell lines; SOS, A2780 after treatment with melittin + cisplatin.

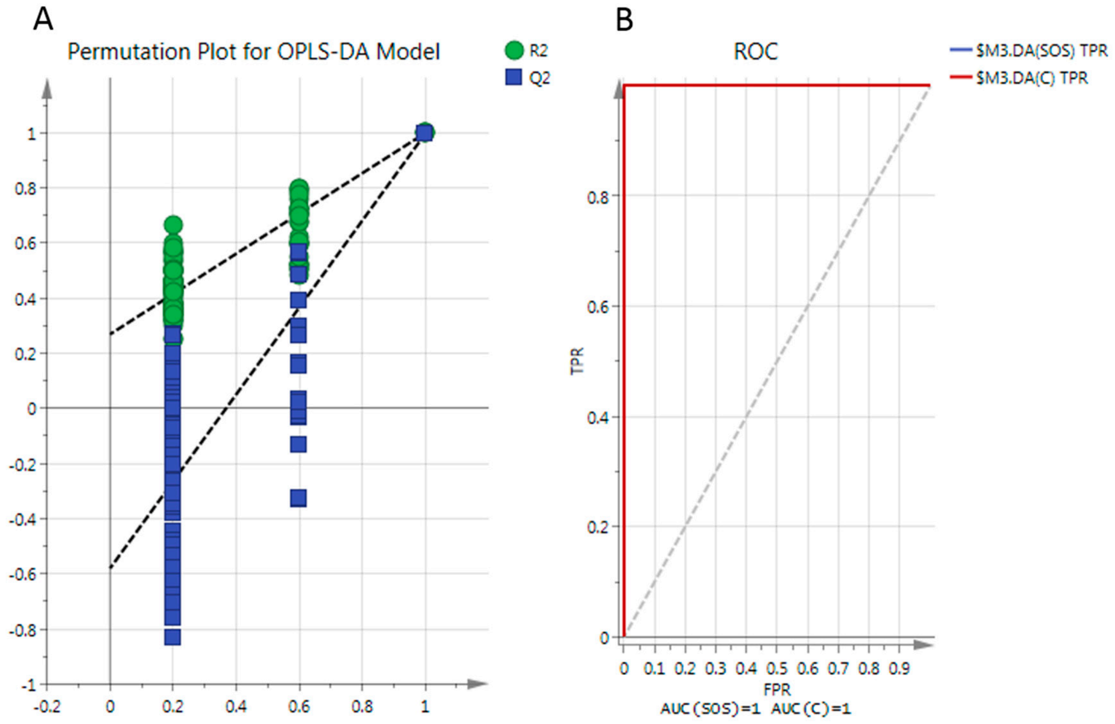

Figure S4 **(A)** Permutation analysis of OPLS-DA model derived from A2780 cells treated with melittin/cisplatin and controls cells. Statistical validation of the OPLS-DA model by permutation analysis using 100 different model permutations. The goodness of fit (R2) and predictive capability (Q2) of the original model are indicated on the far right and remain higher than those of the 100 permuted models to the left. OPLS-DA, orthogonal partial least squares discriminant analysis. **(B)** Receiver Operating Characteristics (ROC) curve shows sensitivity (true positive rate (TPR)) on the y-axis versus (false positive rate (FPR = 1 − Specificity)) on the x-axis. The area under ROC curve (AUROCC) = 1 for SOS and C groups.

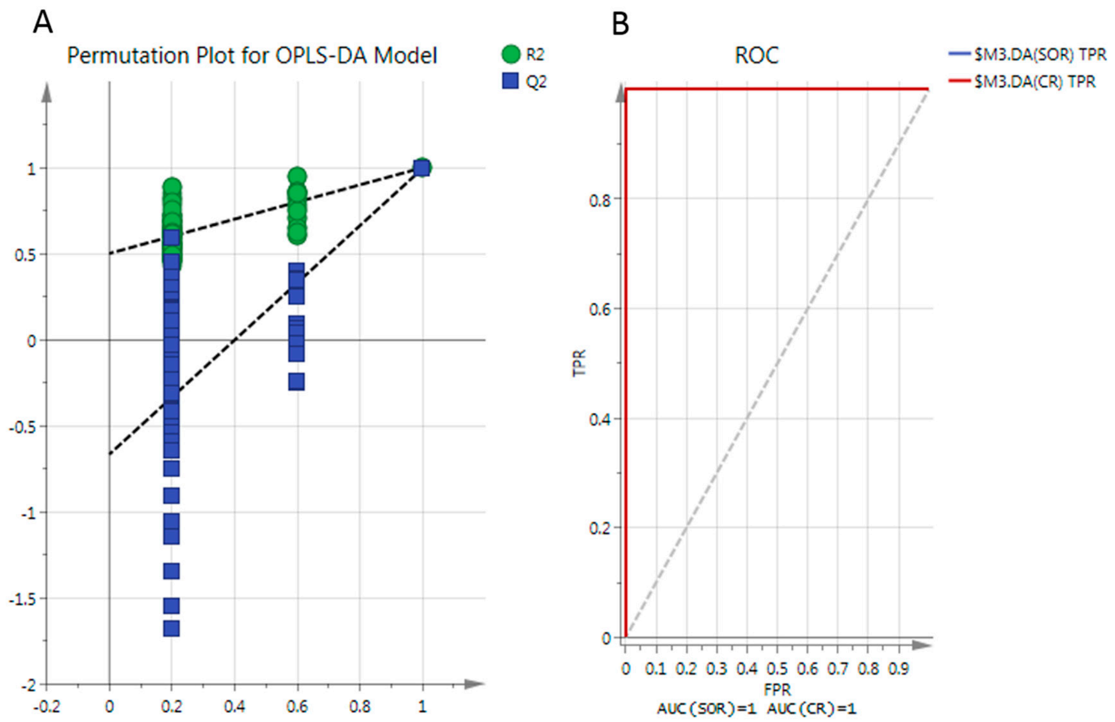

Figure S5 **(A)** Permutation analysis of OPLS-DA model derived from A2780CR cells treated with melittin/cisplatin and controls cells. Statistical validation of the OPLS-DA model by permutation analysis using 100 different model permutations. The goodness of fit (R2) and predictive capability (Q2) of the original model are indicated on the far right and remain higher than those of the 100 permuted models to the left. OPLS-DA, orthogonal partial least squares discriminant analysis. **(B)** Receiver Operating Characteristics (ROC) curve shows sensitivity (true positive rate (TPR)) on the y-axis versus (false positive rate (FPR = 1 − Specificity)) on the x-axis. The area under ROC curve (AUROCC) = 1 for SOR and CR groups.
